# Probing islet stress in type 1 diabetes

**DOI:** 10.18632/aging.104157

**Published:** 2020-10-12

**Authors:** Ernesto S. Nakayasu, Thomas O. Metz, Raghavendra G. Mirmira

**Affiliations:** 1 Biological Sciences Division, Pacific Northwest National Laboratory, Richland, WA 99354, USA; 2 Kovler Diabetes Center and the Department of Medicine, The University of Chicago, Chicago, IL 60637, USA

**Keywords:** diabetes, islet, autoimmunity. proteomics

Type 1 diabetes (T1D) has historically been viewed as a disorder of immune tolerance, whereby autoreactive T cells infiltrate, recognize, and destroy insulin-producing islet β cells. Despite this understanding, immune-targeted therapies in the setting of T1D have neither uniformly nor durably preserved insulin secretory capacity. The picture of immune cell infiltration into islets (known as “insulitis”) was first described in the early 1900s, many years before the discovery of insulin itself. The presence of insulitis has subsequently come to define a pathologic hallmark of T1D. Yet, in recent years the analysis of postmortem tissues from donors with T1D has revealed an unexpected finding: namely, that aggressive T cell-mediated insulitis and rapid loss of β-cell mass has not proved uniformly true in humans. For example, postmortem studies show that fewer than 24% of T1D individuals have any detectable pathologic evidence of insulitis [1], and still other studies show both a pre-diabetic increase in proinsulin/C-peptide ratio and a striking persistence of proinsulin secretion, indicating a preservation of β cells even in longstanding T1D [2]. Likewise, loss of islet β-cell mass displays striking variability, with some individuals exhibiting insulin-positivity in up to 50% of islets at T1D onset [3]. How, then, does one reconcile the notion that aggressive autoimmunity results in the loss of β-cell mass in T1D with the findings that significant numbers of β cells, however hypofunctioning, persist many years after the onset of disease? One perspective posits that T1D is a disease of both autoimmunity and the β cell, where the susceptibility of the latter to the immune-mediated death or dysfunction might play a crucial role in the development of hyperglycemia.

The perspective that the β cell, or a subpopulation thereof, contributes to disease because of differential susceptibility to immune attack is not entirely speculative. Studies of human islets suggest that they contain distinct subpopulations of β cells [4], and recent studies in NOD mice identified subpopulations that resist or invite T cell attack [5]. Taken together, these emerging data imply a strategy wherein therapies that target both the immune response and molecular pathways within the β cell that increase susceptibility to apoptosis represent a more effective approach to prevent or reverse T1D.

Studies of gene expression (microarray analyses, RNA sequencing strategies, and targeted PCR-based approaches) have provided insight into the nature of stress pathways activated in β cells during T1D. Particularly are the molecular nodes that converge on endoplasmic reticulum (ER) stress, a process whereby the capacity of the ER to fold and process proteins is overwhelmed and results in a response that suppresses the translation of mRNAs in the cell [6]. The nature of this stress process implies that the mere study of gene expression may be inadequate to identify crucial proteins and metabolic processes that function at critical junctures of cellular survival vs. apoptosis (since the proteins themselves may be missing owing to the suppression of mRNA translation).

In a recent study [7], our group took a proteomics approach to identify proteins whose under- or over-production might contribute to β-cell susceptibility. Human pancreatic islets were treated *in vitro* with proinflammatory cytokines interleukin-1β (IL-1β) and interferon-γ (IFN-γ), the very same cytokines that have been implicated in the earliest phases of immune cell infiltration in T1D. Among proteins that were differentially altered in response to these cytokines and were classified as regulatory proteins that functioned in apoptosis, we identified secreted phosphoprotein-1 (SPP1) and growth and differentiation factor-15 (GDF15). Of the two, GDF15 levels were reduced despite an upregulation in its mRNA levels—a finding consistent with ER stress-mediated suppression of mRNA translation. We hypothesized that GDF15, a previously identified anti-inflammatory cytokine that induces tissue tolerance, would confer protection against islet apoptosis. Exogenously applied GDF15 prevented apoptosis of human islets exposed to proinflammatory cytokines *in vitro*, and delivery of GDF15 systemically to NOD mice delayed the development of T1D. These findings not only emphasize the potential utility of leveraging proteomics and related strategies for identifying new targets for T1D prevention and therapy, but also emphasize the necessity of utilizing proteomics in the setting of cellular stress, where posttranscriptional regulation renders other strategies potentially misleading.

Notwithstanding our findings, there remain challenges to utilizing proteomics of islets for the discovery of new targets in T1D. First and foremost, our findings emanated from a model of T1D *in vitro*. Disease pathogenesis in humans remains to be fully defined and, as such, models represent only a first approximation to how disease might develop in the early stages. Therefore, in the absence of the technical capability of sampling islets from subjects with active disease (which also faces ethical hurdles), the greatest challenge faced by the field will be obtaining cadaveric islets from individuals at different stages of the disease. New technologies that allow for the proteomics analysis of fixed tissues from such donors (for a review, see [8]) have the potential to reduce the need for fresh islet isolations. Second, a major challenge exists in obtaining sufficient depth of analysis of proteins from small cell populations obtained from donors with disease, although new nano-scale technologies are now being developed. These nano-scale technologies have also enabled the ability to perform imaging proteomics [8], allowing for the study of protein distribution and heterogeneity in tissues in large scale. As we begin to understand better the role of the β cell in the pathogenesis of T1D, the comprehensive molecular analysis of the β cell itself will be necessary in developing next-generation therapies. Such therapies must move beyond simply the knowledge garnered from genomics and functional genomics analyses.

## References

[1] In’t Veld P. Semin Immunopathol. 2014; 36:569–79. 10.1007/s00281-014-0438-4 25005747 PMC4186970

[2] Sims EK, et al. Diabetes Care. 2019; 42:258–64. 10.2337/dc17-2625 30530850 PMC6341288

[3] Campbell-Thompson M, et al. Diabetes. 2016; 65:719–31. 10.2337/db15-0779 26581594 PMC4764143

[4] Dorrell C, et al. Nat Commun. 2016; 7:11756. 10.1038/ncomms11756 27399229 PMC4942571

[5] Rui J, et al. Cell Metab. 2017; 25:727–38. 10.1016/j.cmet.2017.01.005 28190773 PMC5342930

[6] Evans-Molina C, et al. Diabetes Obes Metab. 2013 (Suppl 3); 15:159–69. 10.1111/dom.12163 24003933 PMC3777692

[7] Nakayasu ES, et al. Cell Metab. 2020; 31:363–374.e6. 10.1016/j.cmet.2019.12.005 31928885 PMC7319177

[8] Nakayasu ES, et al. Expert Rev Proteomics. 2019; 16:569–82. 10.1080/14789450.2019.1634548 31232620 PMC6628911

